# Long-term antimicrobial effectiveness of a silver-impregnated foil on high-touch hospital surfaces in patient rooms

**DOI:** 10.1186/s13756-021-00956-1

**Published:** 2021-08-16

**Authors:** Andreas F. Widmer, Sonja Kuster, Marc Dangel, Sammy Jäger, Reno Frei

**Affiliations:** 1 Division of Infectious Diseases and Hospital Epidemiology, University Hospital Basel, 4031 Basel, Switzerland; 2 Present Address: Spital Muri, 5630 Muri, Switzerland

**Keywords:** Healthcare-associated infections, Environment, Surface disinfection, Silver containing foil, Auto-disinfection, Enterococci

## Abstract

**Background:**

The hospital environment has got more attention as evidence as source for bacterial transmission and subsequent hospital-acquired infection increased. Regular cleaning and disinfection have been proposed to lower the risk of infection, in particular for gram-positive bacteria. Auto-disinfecting surfaces would allow to decrease survival of pathogens, while limiting resource to achieve a safe environment in patient rooms.

**Methods:**

A controlled trial to evaluate the antimicrobial effectiveness of a polyvinyl chloride foil containing an integrated silver-based agent (containing silver ions 2%) on high-touch surfaces in patient rooms.

**Results:**

The overall log reduction of the mean values was 1.8 log_10_ CFU, the median 0.5 log_10_ CFU comparing bioburden of control vs antimicrobial foil (p < 0.01). Important pathogens were significantly less likely recovered from the foil, in particular enterococci. These effects were present even after 6 months of in-use.

**Conclusions:**

A foil containing an integrated silver-based agent applied to high-touch surfaces effectively results in lower recovery of important pathogens from such surfaces over a 6-month study period.

**Supplementary Information:**

The online version contains supplementary material available at 10.1186/s13756-021-00956-1.

## Introduction

Healthcare-associated infections (HAIs) affect millions of patients every year challenging healthcare institutions [1]. In Europe, 6.5% of patients in acute care hospitals develop at least one HAI [2]. By effective infection control programs, 20–30% of HAIs are considered to be preventable. Goals to decrease HAIs have been met in the last decade, but a large burden for patients remains still requiring more efforts for prevention [3]. Nosocomial pathogens causing HAIs originate from the patients’ endogenous flora in 40–60%, in 20–40% as results of cross-infection from contaminated hands of healthcare workers (HCW) and approximately 20% from contamination of the environment, depending on the pathogen [4]. Multidrug-resistant microorganisms as well as *Clostridioides difficile* are frequent causes of HAIs, in particular meticillin-resistant *Staphylococcus aureus* (MRSA), vancomycin-resistant enterococci (VRE), and *Acinetobacter baumannii* that are commonly involved in the transmission of these pathogens [5–7]. These microorganisms can persist on hospital surfaces from hours to months, depending on location, number, biofilm formation, intrinsic resistance of organisms to various cleaning products as well as local conditions [8]. For VRE, contact with a contaminated environment results in a similar risk of contamination of HCW hands, independent of contact with intact skin of a colonized patient or his environment [9]. Cleaning and disinfection may reduce HAIs [6, 7], as documented for *C. difficile* [10]. Routine contact isolation for patients with non-hypervirulent *C. difficile* can even be lifted, if standard precautions and cleaning of the environment are ensured [11, 12]. Enhanced cleaning and disinfection can even be cost-effective [13]. Pathogens can be transmitted by hands of HCW after patient care, but also from touching the environment close to patients [9]. Routine hand hygiene would eliminate this risk, but in reality, high compliance and appropriate technique remain an ongoing challenge [14]. Another approach is repetitive daily cleaning and disinfection of critical surfaces, but this is very costly, and can impede patient care. In addition, recolonization of the surfaces occurs within hours after cleaning [15, 16]. Even terminal cleaning after patient discharge cannot always kill pathogens left on surfaces by prior room occupants [17]. Therefore, continuous antimicrobial activity against microorganisms by auto-disinfecting materials or coating are current matter of research [18]. Copper has been successfully tested to prevent HAIs in hospitals, but it is very expensive, heavy and confirmation studies are pending [19]. A very recent clinical trial compared high-touch surfaces coated with a quaternary ammonium polymer with non-coated surfaces to determine the impact on the incidence of HAIs [20]. Similarly, this study tested a silver-impregnated foil mounted on high-touch surfaces in patient rooms, to evaluate the antimicrobial activity on bioburden and presence of important pathogens.

## Materials and methods

### Antimicrobial foil

The commercially available antimicrobial foil PURZON060B produced by HEXIS S.A. (Frontignan, France) was used for the study. The flexible, transparent and 60 µm thick polyvinyl chloride foil contains an integrated silver-based agent (containing 2% silver ions) developed and manufactured by SANITIZED AG (Burgdorf, Switzerland). Detailed specifications of the antimicrobial foil PURZON060B are provided at https://hexis-graphics.com/documents/fichestechnique/document_en/aut_PURZON060B_FTP_anglais.pdf.  Last access June 3, 2021.

### Study setting

The prospective and comparative study was conducted in one surgical and one medical ward at the University Hospital Basel from March through May 2020. Based on a previous study [21], a reduction of > 50% of the bioburden or important pathogens was considered as clinically meaningful.

On each ward, high-touch surfaces in three patient rooms were coated with the antimicrobial foil. The following high touch surfaces were selected: overbed table, nightstand, armrest of a resting chair, dining table, toilet ring and toilet flusher. The corresponding control surfaces were defined on the same furniture, either adjacent or on the other side (e.g. left and right armrest). The right or left position of the foil or control surface was selected alternately. Since the toilet ring and the toilet flusher had to be fully covered with foil for technical reasons, the controls were taken from an adjacent patient room. Overall, 12 overbed tables, 12 nightstands, 8 armrests, 7 dining tables, 4 toilet rings and 4 toilet flushers were coated with antimicrobial foil, resulting in 47 coated test surfaces and 47 uncoated control surfaces. The self-adhesive antimicrobial foil was applied by trained technicians.

### Sampling

Samples for microbiological investigations were collected every Monday and Wednesday after 5 pm with flocked swabs moistened with NaCl solution prior to use and after swabbing put in eSwab® transport medium (Copan, Brescia, Italy). Guided by clean 25.2 cm^2^ metal templates, the test foil as well as the control surfaces were swabbed. The swabs were immediately brought to the microbiology laboratory and stored at 4–8 °C overnight before processing.

### Laboratory methods

Out of the eSwab® fluid, 250 µl were inoculated on each of the following culture media: trypticase soy agar, ChromID® CPS® Elite and ChromID® *S. aureus* Elite (bioMérieux, Marcy-l’Étoile, France). The media were incubated at 36° ± 1 °C for 42–48 h. Colony-forming units (CFU) were counted and suspected pathogenic isolates were identified with matrix-assisted laser desorption/ionization – time of flight (MALDI-TOF) mass spectrometry (MALDI Biotyper®, microflex™ LT/SH smart, Bruker Daltonik, Bremen, Germany). The microbiological analysis focused on the following important pathogens: *S. aureus*, *Enterococcus faecalis*, *E. faecium*, other *Enterococcus* spp., haemolytic streptococci, *Enterobacterales*, *Pseudomonas* spp., and *A*. *baumannii* group.

### Policy of cleaning and disinfection of the environment

All patient rooms are cleaned once daily with a detergent and single-use microfiber pads. Washrooms are routinely disinfected with Deconex® 50FF (Borer Chemie, Zuchwil, Switzerland), an aldehyde-free certified disinfectant based on ethanedial, pentanedial and didecyldimethylammonium chlorid.

### Statistical analysis

Data was collected in a spreadsheet, imported into and analyzed with Python 3.7.7 (pandas 1.0.3, scipy 1.4.1, numpy 1.18.4). Culture results were reported as log_10_ CFU /cm^2^. The mean log_10_ reduction was calculated as difference between the log_10_ of the mean CFU of samples taken from the antimicrobial foil and the control surface. The median log_10_ reduction was calculated respectively. For the comparison between CFU on the antimicrobial foil versus the uncoated control surface, the values were compared by Wilcoxon signed rank test and for nonrelated samples by Mann Whitney U test. P values < 0.05 (two-sided) were considered statistically significant.

## Results

Overall, 403 swabs were sampled: 201 from the antimicrobial foil, 202 from uncoated control surfaces. Cultures were negative in 79 samples: 53 (67%) from antimicrobial foil and 26 (33%) from control samples (p < 0.001). The overall log reduction of the mean values was 1.8 log_10_ CFU, the median 0.5 log_10_ CFU comparing bioburden of control versus antimicrobial foil (p < 0.01, Tables 1, 2). Higher reductions were observed in samples with high bioburden on control samples: The highest reduction was observed in toilet samples with 2.0 log_10_ CFU reduction (p < 0.01).

**Table 1:** Mean log_10_ reduction CFU overall and CFU of important pathogens on antimicrobial foil compared to control surfaces

	N samples	Mean (SD)	p value	Reduction
CFU/cm^2^ overall				
Control surfaces	202	378 (± 3240)	< 0.001	1.8 log_10_ CFU
Antimicrobial foil	201	5.58 (± 24.8)		
CFU/cm^2^ of important pathogens*				
Control surfaces	202	60.61 (± 843.14)	< 0.001	2.6 log_10_ CFU
Antimicrobial foil	201	0.14 (± 0.1.59)		

Very few gram-negative bacteria were isolated to make meaningful comparisons

^*^ > 90% *S. aureus*, *E. facalis* and *E. faecium*, see Materials and methods

**Table 2:** Median log_10_ reduction CFU overall on antimicrobial foil compared to control surfaces

	N samples	Median (interquartile range)	p value	Reduction
CFU/cm^2^ overall				
Control surfaces	202	0.95 (0.16–5.52)	< 0.001	0.5 log_10_ CFU
Antimicrobial foil	201	0.32 (0.02–1.90)		

**Fig. 1:**
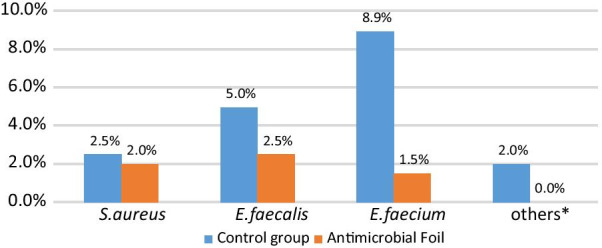
Presence of important pathogens

More importantly, 49 different important pathogens were found on 38 samples: 9 (34%) from the antimicrobial foil, 29 (76%) from the control samples (p < 0.001). Over 90% of important pathogens were gram-positive bacteria, bacteria that survive well on dry surfaces. Large differences between the antimicrobial foil compared to the control surfaces were observed in detection of enterococci (Fig. 1). *Acinetobacter* spp. also belong to bacteria that can survive for prolonged periods of time: However, none of the samples were positive for *Acinetobacter baumanii* group. Very few gram-negative bacteria were isolated to make meaningful comparisons.

The long-term effect over 6 months was confirmed by repeating samples from the antimicrobial foil (Table 3).

**Table 3:** CFU after 6 months of use in patient rooms

	N samples	Mean (SD)	p value	Reduction
CFU/cm^2^ overall				
Control surfaces	38	7.44 (± 3.97)	0.12	0.24 log_10_ CFU
Antimicrobial foil	38	4.24 (± 2.71)		
CFU/cm^2^ of important pathogens*				
Control	38	0.04 (± 0.17)	0.04	> 0.6 log_10_ CFU
Antimicrobial foil	38	0.00(± 0.00)		

Very few gram-negative bacteria were isolated to make meaningful comparisons

^*^ > 90% *S. aureus*, *E. facalis* and *E. faecium*, see Materials and methods

## Discussion

Multiple studies have confirmed the impact of proper removal of environmental pathogens on the incidence of transmission [6, 16, 22], even in randomized controlled clinical trials and it appears to be cost-effective [13]. One very recent study indicates that environmental control in hospitals leads to significantly lower rates of HAIs [20]. The importance of environmental contamination control has increased in healthcare institutions over time. It might be of higher importance to fight against transmission of multidrug-resistant microorganisms than for decreasing HAIs. In this study, bioburden was significantly reduced by the antimicrobial foil: measured as reduction in the mean CFU as well as the median, the latter commonly leading to a lower effect [20]. It also succeeded in significant decrease of important pathogens, in particular *E. faecalis* and *E. faecium*. Prior environmental contamination of hospital rooms may increase the risk of acquisition of enterococci [23] and was responsible for one of the largest country-wide epidemic with VRE in Switzerland [24]. The *E. faecium* clone ST 796 was likely introduced from Australia, spread to many Swiss hospitals and lasted more than two years despite intensified contact isolation, preemptive isolation, admission screening and even hospital wide screening of all patients. An effective disinfectant rapidly kills enterococci including VRE, but environmental recolonization occurs within hours after disinfection [15]. Therefore, an auto-disinfectant surface would be desirable in certain areas such as transplant units or also during pandemics as currently with SARS-CoV-2 [25], as bacterial communities seem to contribute to viral prevalence in the hospital environment [26]. The PURZON060B antimicrobial foil has been tested against human coronavirus HCoV-229E in vitro, and was highly active in-vitro (data from a certificate, https://catalogues.hexis-graphics.com/c/frxfr-hexis-pure-zone), supplementary appendix file 1). However, our study design was submitted in autumn 2019, and resources and biosafety limitations precluded testing the product in patient rooms.

The foil could be placed on different surfaces without getting loose over time. However, the removal of the foil needs special expertise, since it sticks very well to the surface. In addition to the impregnated foil, the silver-based antimicrobial compound has been successfully incorporated into a variety of other materials such as fabrics and synthetics to finish various surfaces and equipment making this compound of interest for further applications.

Several study limitations need to be considered: We took samples 8–10 h after routine cleaning and disinfection, after patients and HCW were using the environment as deemed necessary for the daily work and requirements. The frequency of touching surfaces was beyond control of the study, but the hospital policy requires daily cleaning and/or disinfection. However, the sampling technique was designed to take samples exactly from the adjacent area as the antimicrobial foil was put on. A long-term effect over years was not assessed to study longevity. Since during the study the pandemic with SARS-CoV-2 spread throughout Switzerland, the study had to be temporarily interrupted from mid-March to end of April. We continued the study to estimate the effect of massively increased disinfection policy. Due to the lower occupancy of the wards and restricted access of visitors, the bioburden on the study surfaces had significantly decreased to nearly undetectable levels (data not shown). Increased cleaning and disinfection to at least once daily, with emphasis to washrooms could be an effective alternative to the use of such an auto-disinfectant antimicrobial foil.

In conclusion, this polyvinyl chloride foil containing an integrated silver-based agent applied to high-touch surfaces effectively results in lower recovery of important pathogens from such surfaces, even six months after clinical use in patient rooms. Auto-disinfectant foils or similar antimicrobially equipped surfaces might help to prevent transmission—in particular—of gram-positive pathogens from the environment.

## Supplementary Information

**Additional file 1.Appendix 1** (data from a certificate, https://catalogues.hexis-graphics.com/c/frxfr-hexis-purezone), supplementary appendix file 1).

## Data Availability

Data are available as Excel file und python statistical software. The datasets used and/or analysed during the current study are available from the corresponding author on reasonable request.
