# Supplementary material for: Long-term antimicrobial effectiveness of a silver-impregnated foil on high-touch hospital surfaces in patient rooms
Source: Antimicrob Resist Infect Control. 2021 Aug 16;10:120. doi: 10.1186/s13756-021-00956-1 (PMC8365124; doi:10.1186/s13756-021-00956-1)
Supplement: Supplementary file 1 — Additional file 1.Appendix 1 (data from a certificate, https://catalogues.hexis-graphics.com/c/frxfr-hexis-purezone), supplementary appendix file 1). [file 13756_2021_956_MOESM1_ESM.pdf]

Test report R2010FSSAN003
Antiviral activity of 1%BC A 21-41 surface on human coronavirus HCoV-229E for a contact time of 120 minutes.
Adapted protocol from ISO 21702 (2019) standard

**CLIENT**	SANITIZED AG<br>Monsieur Frédéric LOYRION<br>Lyssachstrasse 95<br>3401 Burgdorf<br>Switzerland
**TEST LABORATORY**	S.A.S VIRHEALTH<br>Site Laennec-La Buire, 2éme étage, Bat B<br>7-11 rue Guillaume Paradin,<br>69372 Lyon Cedex 08<br>FRANCE
**TECHNICAL CONTRIBUTION**	Léa Szpiro, technical manager<br>Loranne Durimel, laboratory technicien

QUALITY APPROVAL
Name : Dr Vincent MOULES, CEO
Date : Lyon, 07/10/2020
Signature : 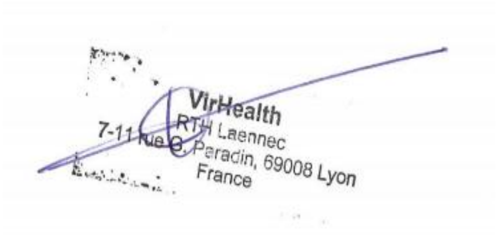

This report includes 12 pages

REPRODUCTION AND DIFFUSION ARE FORBIDDEN WITHOUT PRIOR AUTHORISATION

SUMMARY

I. CONCLUSION ... 3

II. CONTRACTUAL DOCUMENTS ... 4

III. TEST CONDITIONS AND SAMPLES DATA ... 4

III.1 SAMPLES IDENTIFICATION ... 4

III.2 EXPERIMENTAL CONDITIONS ... 5

IV. RESULTS ... 6

ANTIVIRAL ACTIVITY OF THE 1%BC A 21-41 SURFACE ON HUMAN CORONAVIRUS HCOV-229E FOR A CONTACT TIME OF 120 MINUTES ... 6

a. Cell susceptibility ... 6

b. Cytotoxicity ... 6

c. Inactivation of antiviral activity ... 7

d. Test ... 8

V. CONCLUSION ... 9

VI. ANNEXES ... 10

VI.1. MATERIALS AND REAGENTS ... 10

VI.2 RAW DATA: $TCID_{50}$ QUANTIFICATION OF HUMAN CORONAVIRUS HCOV-229E AFTER 120 MINUTES, VISUAL READING OF CYTOPATHIC EFFECTS (8 WELLS PER DILUTIONS) ... 11

REPRODUCTION AND DIFFUSION ARE FORBIDDEN WITHOUT PRIOR AUTHORISATION

I. CONCLUSION

Antiviral activity of the 1% BC A 21-41 surface and non-active surface has been tested under conditions defined by the ISO 21702 (2019) adapted protocol for contact times of 120 minutes on the human coronavirus HCoV-229E.

The reference surface (stainless steel) is the control for this test.

- 1%BC A 21-41 surface, 120 minutes of contact time

Under experimental conditions, (20°C, 120 minutes), the 1% BC A 21-41 surface shows an antiviral activity per cm^2 associated with a logarithmic reduction of 1.4 log_{10} (96.02%) under the ISO 21702 adapted protocol.

PRODUCT	Contact time (min)	Antiviral activity R (log_{10}/cm^2)	Antiviral activity (%)
1%BC A 21-41	120	**R = 1.4**	96.02

REPRODUCTION AND DIFFUSION ARE FORBIDDEN WITHOUT PRIOR AUTHORISATION

II. CONTRACTUAL DOCUMENTS

The present service is defined by the following contractual documents:

**. Quotation**	DEV0100 from 23/07/2020
**. Order**	Good for agreement from 03/09/2020

III. TEST CONDITIONS AND SAMPLES DATA

III.1 Samples identification

Test surface	**1%BC A 21-41**	**INOX**
Appearance	White	Grey (stainless steel)
Size (cm/cm²)	5cm x 5cm / 25 cm²	5cm x 5cm / 25 cm²
Thickness (mm)	1	2
Porous/ non porous	Non porous	Non porous

Manufacturer: SANITIZED AG

Supplier: SANITIZED AG

Storage conditions: room temperature

Evaluation period: 10/2020

REPRODUCTION AND DIFFUSION ARE FORBIDDEN WITHOUT PRIOR AUTHORISATION

III.2 Experimental conditions

Test surface: 1%BC A 21-41

Experimental Conditions	
Date	01/10/2020
Viral strain	Human coronavirus HCoV-229E
Inoculum size (cm²)	4 cm x 4 cm = 16 cm²
Inoculum volume	400µL
Cover film	n.a.
Temperature	20°C
Humidity HR (%)	46%
Contact time	120 minutes
Interfering substance	n.a.
Neutralisation	10mL of SCDLP medium
Quantification	endpoint titration on permissives cells
Number of wells per dilution	8
Incubation temperature	34 ± 1 °C

REPRODUCTION AND DIFFUSION ARE FORBIDDEN WITHOUT PRIOR AUTHORISATION

IV. RESULTS

Antiviral activity of the 1%BC A 21-41 surface on human coronavirus HCoV-229E for a contact time of 120 minutes

a. Cell susceptibility

Product	Log_{10} $TCID_{50}$/mL
SCDLP medium	6.6
Active surface	6.7
Non active surface (reference)	6.7
Active surface: Difference < 0.5 log_{10} ☒ yes ☐ no<br>Non-active surface: Difference < 0.5 log_{10} ☒ yes ☐ no	

b. Cytotoxicity

The test surface cytotoxicity is determined by reading of cytopathic effect (CPE) on MRC5 permissive cells and quantified by $TCID_{50}$ technique.

For viral recuperation on surface, the surfaces are submerging in 10mL of SCDLP medium (recuperation buffer). The recuperation buffer cytotoxicity is determined by reading of cytopathic effect (CPE).

Under test conditions, the recuperations buffers from 1%BC A 21-41 and reference surfaces did not show cytopathic effects on MRC5 cells for a contact time of 120 minutes.

The test results are dependent on and take into account the cytotoxicity results.

REPRODUCTION AND DIFFUSION ARE FORBIDDEN WITHOUT PRIOR AUTHORISATION

c. Inactivation of antiviral activity

Product	Log_{10} $TCID_{50}$/mL
S_n = SCDLP medium	5.9
S_t = Active surface	6.1
S_u = Non active surface (reference)	6.2
$S_n - S_u \leq 0,5\ log_{10}$ ☒ yes ☐ no<br>$S_n - S_t \leq 0,5\ log_{10}$ ☒ yes ☐ no	

Explanations:

S_n: the average of the common logarithm of the infectivity titer of virus from three of the SCDLP broth for negative control.

S_u: the average of the common logarithm of the infectivity titer of virus recovered from three of the untreated test specimens;

S_t: the average of the common logarithm of the infectivity titer of virus recovered from three of the test specimens.

By comparing the infectivity titer of virus from the SCDLP broth for negative control with that from the untreated test specimen and the treated specimen, we observe a logarithmic reduction of less than 0.5 Log_{10}.

REPRODUCTION AND DIFFUSION ARE FORBIDDEN WITHOUT PRIOR AUTHORISATION

d. Test

Raw data for antiviral activity of 1%BC A 21-41 and reference surfaces on human coronavirus HCoV-229E under test conditions (20°C, 120 minutes) are presented in appendices.

Results have been determined by visual reading of cytopathic effects (CPE) and quantified by $TCID_{50}$ technique on MRC5 cells.

Surface	Cytotoxicity (log_{10} $TCID_{50}$)	Specimen	U_o (log_{10} $TCID_{50}/cm^2$)	U_{t120} (log_{10} $TCID_{50}/cm^2$)
**Reference surface stainless steel**	0.5	L1	6,1	5,9
		L2	5,8	6,5
		L3	6,4	5,9
		Average	**6,1**	**6,1**

Surface	Cytotoxicity (log_{10} $TCID_{50}$)	Specimen	A_0 (log_{10} $TCID_{50}/cm^2$)	A_{t120} (log_{10} $TCID_{50}/cm^2$)
**1%BC A 21-41**	0.5	L1	5,9	4,5
		L2	5,9	5
		L3	6	4,7
		Average	**5,9**	**4,7**
		R (log_{10} $TCID_{50}/cm^2$)	/	**1.4**

R is the antiviral activity
U_0 is the average of the common logarithm of the number of plaques recovered from three untreated test specimens immediately after inoculation
U_t is the average of the common logarithm of the number of plaques recovered from three untreated test specimens
A_0 is the average of the common logarithm of the number of plaques recovered from three treated test specimens immediately after inoculation
A_t is the average of the common logarithm of the number of plaques recovered from three treated test specimens.

The logarithmic value of the number of $TCID_{50}$ recovered immediately after inoculation from the test specimens satisfies the requirement below: $(L_{max} - L_{min}) / (L_{mean}) \leq 0.2$.

V. CONCLUSION

1%BC A 21-41 surface shows antiviral activity of 1.4 log_{10} $TCID_{50}/cm^2$ (96.02%) on human coronavirus HCoV-229E after a contact time of 120 minutes at 20°C.

REPRODUCTION AND DIFFUSION ARE FORBIDDEN WITHOUT PRIOR AUTHORISATION

VI. ANNEXES

VI.1. Materials and reagents

- Cell line

Name : MRC5 ATCC® CCL-171™

Number of passages : 19

Culture medium: EMEM (Lonza, batch n°0000757679, 11/2020) with 10% of FCS (Dutscher, batch n° S16529S1810, 09/2022), 1% of antibiotics (Gibco, batch n° 2145466, 12/2020) and 1% of L-glutamine (Gibco, batch n° 2091579, 22/2021)

- viral strain

name: human coronavirus 229E ATCC® VR-740™

Viral suspension test: 2.37x10^7 (batch number: 072020229-2)

Quantification:

- tenfold dilution in infection medium : EMEM (Lonza, batch n°0000757679, 11/2020) with 2% of SVF (Dutscher, batch n° S16529S1810, 09/2022), 1% of antibiotics (Gibco, batch n° 2145466, 12/2020) and 1% of L-glutamine (Gibco, batch n° 2091579, 22/2021)
- add 100ul in a 96 wells plates
- Incubate 7 days at 34°C, 5% of $C0_2$

REPRODUCTION AND DIFFUSION ARE FORBIDDEN WITHOUT PRIOR AUTHORISATION

VI.2 RAW DATA: $TCID_{50}$ quantification of human coronavirus HCoV-229E after 120 minutes, visual reading of cytopathic effects (8 wells per dilutions)

- Table 1: cell susceptibility

	Product	Contact time (min)	dilutions (-log)							
			P	1	2	3	4	5	6	7
sensibility	SCDLP medium	/	44444444	44444444	44444444	44444444	44444444	10000000	0	0
	Untreated	/	44444444	44444444	44444444	44444444	44444444	20030000	0	0
	1%BC A 21-41	/	44444444	44444444	44444444	44444444	44444444	10001000	0	0

Explanations:
- *1-4: degrees of CPE in 8 cell culture unit (microtiter plate)*
- *0: no virus present*
- *n.a: not applicable*
- *n.d: not done*

- Table 2: control of suppression of antiviral activity

	Product	Contact time (min)	dilutions (-log)							
			P	1	2	3	4	5	6	7
Supression of product's activity	SCDLP medium	0	44444444	44444444	44444444	44444444	10023022	0	0	0
		0	44444444	44444444	44444444	44444444	22000000	0	0	0
		0	44444444	44444444	44444444	44444444	211400000	0	0	0
	1%BC A 21-41	0	44444444	44444444	44444444	44444444	02200012	0	0	0
		0	44444444	44444444	44444444	44444444	01121001	0	0	0
		0	44444444	44444444	44444444	44444444	22340012	0	0	0
	Untreated	0	44444444	44444444	44444444	44444444	00244200	0	0	0
		0	44444444	44444444	44444444	44444444	11232002	0	0	0
		0	44444444	44444444	44444444	44444444	01224230	0	0	0

Explanations:
- *1-4: degrees of CPE in 8 cell culture unit (microtiter plate)*
- *0: no virus present*
- *n.a: not applicable*
- *n.d: not done*

- Table 3 : cytotoxicity

	Product	Contact time (min)	dilutions (-log)							
			P	1	2	3	4	5	6	7
cytotoxicity	Untreated	120	0	0	0	0	0	0	0	0
			0	0	0	0	0	0	0	0
			0	0	0	0	0	0	0	0
	1%BC A 21-41	120	0	0	0	0	0	0	0	0
			0	0	0	0	0	0	0	0
			0	0	0	0	0	0	0	0

Explanations:
- *1-4: degrees of CPE in 8 cell culture unit (microtiter plate)*
- *0: no virus present*
- *n.a: not applicable*
- *n.d: not done*

REPRODUCTION AND DIFFUSION ARE FORBIDDEN WITHOUT PRIOR AUTHORISATION

- Table 4 : A_0/U_0

	Product	Contact time (min)	dilutions (-log)							
			P	1	2	3	4	5	6	7
A0/U0	1%BC A 21-41	0	44444444	44444444	44444444	44444444	23020000	0	0	0
		0	44444444	44444444	44444444	44444444	22100000	0	0	0
		0	44444444	44444444	44444444	44444444	10002301	0	0	0
	Untreated	0	44444444	44444444	44444444	44444444	10024440	0	0	0
		0	44444444	44444444	44444444	44444444	00002300	0	0	0
		0	44444444	44444444	44444444	44444444	02222222	0	0	0

Explanations:
- *1-4: degrees of CPE in 8 cell culture unit (microtiter plate)*
- *0: no virus present*
- *n.a: not applicable*
- *n.d: not done*

- Table 5 : test

	Product	Contact time (min)	dilutions (-log)							
			P	1	2	3	4	5	6	7
Test	Untreated	120	44444444	44444444	44444444	44444444	10001100	0	0	0
			44444444	44444444	44444444	44444444	11111111	0	0	0
			44444444	44444444	44444444	44444444	11010000	0	0	0
	1%BC A 21-41		44444444	44444444	44444444	0	0	0	0	0
			44444444	44444444	44444444	10101100	0	0	0	0
			44444444	44444444	44444444	01000100	0	0	0	0

Explanations:
- *1-4: degrees of CPE in 8 cell culture unit (microtiter plate)*
- *0: no virus present*
- *n.a: not applicable*
- *n.d: not done*

REPRODUCTION AND DIFFUSION ARE FORBIDDEN WITHOUT PRIOR AUTHORISATION
